# Supplementary material for: Evolution of stickleback spines through independent cis-regulatory changes at HOXDB
Source: Nat Ecol Evol. 2022 Sep 1;6(10):1537–52. doi: 10.1038/s41559-022-01855-3 (PMC9525239; doi:10.1038/s41559-022-01855-3)
Supplement: Supplementary file 1 — Reporting Summary [file 41559_2022_1855_MOESM1_ESM.pdf]

nature portfolio

Corresponding author(s): David M. Kingsley

Last updated by author(s): 06/09/2022

Reporting Summary

Nature Portfolio wishes to improve the reproducibility of the work that we publish. This form provides structure for consistency and transparency in reporting. For further information on Nature Portfolio policies, see our Editorial Policies and the Editorial Policy Checklist.

Statistics

For all statistical analyses, confirm that the following items are present in the figure legend, table legend, main text, or Methods section.

n/a	Confirmed	
☐	☒	The exact sample size (n) for each experimental group/condition, given as a discrete number and unit of measurement
☐	☒	A statement on whether measurements were taken from distinct samples or whether the same sample was measured repeatedly
☐	☒	The statistical test(s) used AND whether they are one- or two-sided<br>*Only common tests should be described solely by name; describe more complex techniques in the Methods section.*
☐	☒	A description of all covariates tested
☐	☒	A description of any assumptions or corrections, such as tests of normality and adjustment for multiple comparisons
☐	☒	A full description of the statistical parameters including central tendency (e.g. means) or other basic estimates (e.g. regression coefficient) AND variation (e.g. standard deviation) or associated estimates of uncertainty (e.g. confidence intervals)
☐	☒	For null hypothesis testing, the test statistic (e.g. F, t, r) with confidence intervals, effect sizes, degrees of freedom and P value noted<br>*Give P values as exact values whenever suitable.*
☒	☐	For Bayesian analysis, information on the choice of priors and Markov chain Monte Carlo settings
☒	☐	For hierarchical and complex designs, identification of the appropriate level for tests and full reporting of outcomes
☐	☒	Estimates of effect sizes (e.g. Cohen's d, Pearson's r), indicating how they were calculated

Our web collection on statistics for biologists contains articles on many of the points above.

Software and code

Policy information about availability of computer code

Data collection	See below.
Data analysis	The custom code, programs (versions and associated parameters) used to analyze the data is available at figshare and details are provided in the materials and methods.

For manuscripts utilizing custom algorithms or software that are central to the research but not yet described in published literature, software must be made available to editors and reviewers. We strongly encourage code deposition in a community repository (e.g. GitHub). See the Nature Portfolio guidelines for submitting code & software for further information.

Data

Policy information about availability of data

All manuscripts must include a data availability statement. This statement should provide the following information, where applicable:

- Accession codes, unique identifiers, or web links for publicly available datasets
- A description of any restrictions on data availability
- For clinical datasets or third party data, please ensure that the statement adheres to our policy

The raw and processed RNA-sequencing data in this paper are available in the NCBI GEO database: GSE184888 (subseries GSE184885, GSE184886, GSE184887, GSE190498). The PacBio HiFi and 10X linked read data from Gasterosteus high-spine sequencing are available in the NCBI databases under BioProject number: PRJNA766710. The 10X linked read data from Apeltes quadracus four- and five-spine fish are available under BioProject number: PRJNA769115. The sequence surrounding AxE in Gasterosteus from the two parental QTL populations and the Apeltes AxE sequences tested in transgenic assays are available in GenBank (OK383406, OK383407, OK383404, OK383405). QTL mapping files, phenotype data files, association mapping genotype files, and code are available at Figshare. The pTia1l-hspGFP plasmid is available from Addgene. Other materials will be made available upon request.

Field-specific reporting

Please select the one below that is the best fit for your research. If you are not sure, read the appropriate sections before making your selection.

☐ Life sciences ☐ Behavioural & social sciences ☒ Ecological, evolutionary & environmental sciences

For a reference copy of the document with all sections, see nature.com/documents/nr-reporting-summary-flat.pdf

Ecological, evolutionary & environmental sciences study design

All studies must disclose on these points even when the disclosure is negative.

Study description	We conducted QTL mapping in threespine sticklebacks (Gasterosteus aculeatus) and association mapping in four spine sticklebacks (Apeltes quadracus) for phenotypes relating to the axial patterning of sticklebacks including the dorsal spines. We focused on the role of one Hox cluster and examined the gene expression changes that affect the patterning of these axial traits.
Research sample	We collected threespine sticklebacks (Gasterosteus aculeatus) and fourspine stickleback (Apeltes quadracus) from the populations described in Supplementary table 1. All fish raised in the animal facility at Stanford were treated in accordance with the recommendations in the Guide for the Care and Use of Laboratory Animals of the National Institutes of Health, using protocols approved by the Institutional Animal Care and Use Committee of Stanford University (IACUC protocol #13834), in animal facilities accredited by the Association for Assessment and Accreditation of Laboratory Animal Care International (AAALAC).
Sampling strategy	We collected fish that represented the whole range of phenotypes present in the population. When choosing fish to use for the QTL cross, fish with the most extreme phenotypes that were sexually mature were chosen. For RNA-sequencing and expression experiments, fish were chosen based on their reproductive condition and their genotype at the locus of interest.
Data collection	Field sampling was conducted by JIW, TRH, ALH, TER, MAB, and ACD with minnow traps at the locations detailed in supplemental table 1. The QTL mapping genotype data was collected by TRH. All RNA-sequencing data was collected by JIW. Whole genome DNA sequencing data was collected by JIW and EHA.
Timing and spatial scale	Samples for Gasterosteus QTL mapping were collected in 2009; Samples for Apeltes association mapping were collected in 2018 and 2019. Sequence and phenotypic analyses was performed between 2011 and 2021.
Data exclusions	Individuals from the Gasterosteus QTL mapping were excluded from the downstream analysis based on the quality of the genotyping calls from the SNP Chip data as detailed in the materials and methods under "QTL mapping".
Reproducibility	Data analysis is fully reproducible and all raw genomic data, phenotypic measurements, processed files, parameters, and code necessary are provided as supplementary material.
Randomization	For morphological association studies, wild caught fish were assigned to high-spined or low-spined groups based on counting spines in skeletal X-rays. Animal numbering within groups was random, and all animals were include unless they showed evidence of broken spines that would preclude accurate measurements.
Blinding	Phenotype measurements for QTL mapping and association mapping were performed without knowing the genotype information for the given individual.

Did the study involve field work? ☒ Yes ☐ No

Field work, collection and transport

Field conditions	Samples were collected in the late spring and early summer when the fish were in reproductive condition. They were caught using minnow traps, seine nets, or dip nets depending on which was appropriate and approved under the relevant permit.
Location	The locations and species sampled are provided in supplemental table 1.
Access & import/export	Fieldwork has been conducted in compliance with appropriate national laws of each country (USA and Canada) and using permits obtained by the appropriate regional or state authorities.
Disturbance	No disturbance was caused by this study.

Reporting for specific materials, systems and methods

We require information from authors about some types of materials, experimental systems and methods used in many studies. Here, indicate whether each material, system or method listed is relevant to your study. If you are not sure if a list item applies to your research, read the appropriate section before selecting a response.

Materials & experimental systems

n/a	Involved in the study
☒	☐ Antibodies
☒	☐ Eukaryotic cell lines
☒	☐ Palaeontology and archaeology
☐	☒ Animals and other organisms
☒	☐ Human research participants
☒	☐ Clinical data
☒	☐ Dual use research of concern

Methods

n/a	Involved in the study
☒	☐ ChIP-seq
☒	☐ Flow cytometry
☒	☐ MRI-based neuroimaging

Animals and other organisms

Policy information about studies involving animals; ARRIVE guidelines recommended for reporting animal research

Laboratory animals	Wild sticklebacks were collected in the field, and their offspring were raised in the animal facility at Stanford University.
Wild animals	Wild sticklebacks were captured using minnow traps, dip nets, or small minnow seines. The populations used for this study and their GPS coordinates are listed in Table S1.
Field-collected samples	Fish husbandry was done using standard methods for sticklebacks. The fish are housed in the animal facility at Stanford under controlled temperature and lighting conditions. The fish are feed twice daily with a mix of blood worms, mysis shrimp, cyclops, and artemia, and the water quality and health checks are performed daily.
Ethics oversight	All sticklebacks were treated in accordance with the recommendations in the Guide for the Care and Use of Laboratory Animals of the National Institutes of Health, using protocols approved by the Institutional Animal Care and Use Committee of Stanford University (IACUC protocol #13834), in animal facilities accredited by the Association for Assessment and Accreditation of Laboratory Animal Care International (AAALAC).

Note that full information on the approval of the study protocol must also be provided in the manuscript.
